# Supplementary material for: Persuasive linguistic tricks in social media marketing communication—The memetic approach
Source: PLoS One. 2021 Jul 1;16(7):e0253983. doi: 10.1371/journal.pone.0253983 (PMC8248608; doi:10.1371/journal.pone.0253983)
Supplement: Attachment — Submitted filename: 1.docx [file pone.0253983.s001.docx]

**Introduction**

• A concise introduction is required to enable the reader’s understanding of the research problem.

• Expand to emphasize the problem leading to a clear set of research questions and objectives the research addresses.

**Literature review**

Use sub-headings to organize topics. Some critical studies are not included. The paper should relate coherently and convincingly with issues of real-world significance. This is a crucial phase contributing to research design. The theoretical framework emerging from the literature review could research questions and points of emphasis.

**Suggestions**

• Consider summarizing the text based on the purpose of the study.

• I wouldn’t recommend differentiating between the “main” aim and the “specific” aim as is presented in the manuscript, as it might be confusing to the reader.

• Focus more on the empirical studies’ backgrounds.

• Add more information to enable readers’ understanding of the authors’ view.

• You are encouraged to write concisely. The text can be reduced significantly.

**Findings and discussion**

Needs clear and comprehensive explanations to assist readers’ understanding.

**Limitations**

There is no mention of the limitations of this study.

**Summary**

The study presented an important topic that would be of interest to the readership of this journal. Most research is relevant, including to the international audience. However, the research is missing a level of detail needed to understand the study result, the impact of the results, and the research contribution. Perhaps the authors are likely so close to the topic they are skipping over details that they know, but the reader would not.

Overall, the paper requires more focus. The areas requiring attention are highlighted in the individual sections. In summary, the paper needs:

• A re-write of the abstract to give a good summary of the paper and mention the key concepts.

• Expanding the introduction by clearly stating the research problem to suitably inform the reader.

• A synthesized and structured critique of the literature.

• Clarifying the research procedures with an adequate explanation of the methods.

• Expanding the discussion to allow writing a well-developed conclusion summarizing the entire paper. The outcomes should be discussed in relation to the existing research.

• Emphasizing the significance of the research – a clear showing of how the findings contribute to new knowledge.

• Using results to support the claims in conclusion adequately, and how the results of the research can be used for future research.

• The authors are encouraged to write concisely. The text can be reduced significantly.

• In general, the language in the present manuscript is not of publication quality and requires improvement. Please carefully proof-read and spell-check to eliminate grammatical errors.

• The manuscript contains multiple stylistic, grammatical and conceptual flaws. The authors should make sure to differentiate between hyphens and dashes, look into the grammar more carefully (mistakes include “It consist” instead of “It consists”, “Sections 5” instead of “Section 5” and many others), revise intext references (at least one of them is included in the text in full).
