# Supplementary material for: Persuasive linguistic tricks in social media marketing communication—The memetic approach
Source: PLoS One. 2021 Jul 1;16(7):e0253983. doi: 10.1371/journal.pone.0253983 (PMC8248608; doi:10.1371/journal.pone.0253983)
Supplement: Attachment — Submitted filename: Response to Reviewers_2.docx [file pone.0253983.s003.docx]

Dear Reviewers,

thank you for your time and engagement which, probably helps me to improve our manuscript. All of your remarks were relevant and were implemented in the current version of paper. Below, we want to briefly explain some of the improvements in our article according to your feedback. Our answers are marked in blue.

Reviewer #2: Concerning my first remark about missing literature, the authors included in the paper the works I suggested. However, in the introduction they still claim that "the issues of persuasive linguistic tricks and their use in social media marketing communication are rarely found in the scientific literature". My personal opinion is that this claim is not true.

Thank you for this remark. Indeed, that statement went too far. The present version emphasizes the presence in the article of a new, interdisciplinary and evolutionary approach to the issues of linguistic persuasion in SNS.

Second remark about the choice of the typologies of linguistic tricks uniquely based on English literature. Even if I'm not a Polish speaker, of course I agree that the English phrases have an equivalent in Polish. Still, I'm quite sure that there are many other native-Polish phrases that you are missing in your research since it is grounded on English phrases.

Thank you for this remark. The issue of adapting the typology used to the specificity of national languages has been emphasized in the " Limitations of the Study and Future Research" section.

I appreciate that the authors took into consideration my remarks about the description of results. However, I still do not find where the authors illustrate what the "elements of content" are. Unless, the authors refer to the linguistic phrases as "elements of content". I think that this issue should be clarified before the publication.

The phrase "element of content" was used interchangeably by the authors with a "Facebook post". It has been removed and replaced with a simple "post(s)".

I think that the quality of the paper was generally improved after the first round of revisions. However, I think that some minor revisions are needed before publication. The authors should highlighted that they obtained very low correlations, considering all the variables. This should be more stressed. I'm not claiming that it is not a valuable result, but I think it should discussed more in detailed.

Thank you. The issue of low values of correlation coefficients was additionally discussed in „Discussion” section.

Minor remarks: check carefully the direction of the p-value, it is not consistent through the paper. It should be < but in some cases you wrote >.

Those errors have been fixed.

Thank you,

Authors
